# Amplified surface temperature response of cold, deep lakes to inter-annual air temperature variability

**DOI:** 10.1038/s41598-017-04058-0

**Published:** 2017-06-23

**Authors:** R. Iestyn Woolway, Christopher J. Merchant

**Affiliations:** Department of Meteorology, University of Reading, Reading, UK

## Abstract

Summer lake surface water temperatures (LSWTs) have previously been shown to respond more rapidly to climatic warming compared to local summer surface air temperatures (SATs). In a global-scale analysis, we explore the factors underpinning the observation of an amplified response of summer LSWT to SAT variability using 20 years of satellite-derived temperatures from 144 lakes. We demonstrate that the degree of amplification in inter-annual summer LSWT is variable, and is greater for cold lakes (e.g. high latitude and high altitude), which are characterised by a short warming season, and deep lakes, that exhibit long correlation timescales of temperature anomalies due to increased thermal inertia. Such lakes are more likely to display responses in excess of local inter-annual summer SAT variability. Climatic modification of LSWT has numerous consequences for water quality and lake ecosystems, so quantifying this amplified response at a global scale is important.

## Introduction

Climate change is occurring globally and is a first-order control that can affect lakes through a complex series of indirect mechanisms, via effects on the catchment, and direct mechanisms, such as altered thermal and hydrological budgets^1, 2^. An important primary response of a lake to climatic warming is change in lake surface water temperature (LSWT). Change in LSWT causes secondary effects, as temperature is one of the most fundamental drivers of ecosystem structure and function. Temperature affects rates and equilibrium positions of chemical reactions^3^ and rates of metabolic processes^4–6^; it has a pervasive effect on a range of physical, chemical and biological attributes and processes, and influences rates of photosynthesis and respiration^7^, biological growth rates^8^, and organism size^9^. An understanding of LSWT variation and its controls is therefore paramount to understanding how lakes will respond to climate change.

LSWTs can be measured from satellite observations^10, 11^ and can provide detailed information on climate-induced changes in lakes at regional and global scales^12, 13^. Recent efforts have collated satellite data with various *in situ* measurements, to investigate global patterns of summer (July-September) LSWT changes^14^. A recent global synthesis of collated summer LSWT measurements demonstrated that lakes have been warming in recent years, with LSWTs in some regions exceeding nearby surface air temperature (SAT) changes^15^. The amplification of LSWT response to SAT is unexpected from studies of lake surface heat budgets^16^, but several causes could explain the differential in warming, including changes to large-scale climatic forcing^17^ (e.g. solar radiation), an increase or decrease in lake water clarity^18, 19^, and changes to internal lake processes^20^ (e.g. the timing of stratification).

At a global scale, ice-covered lakes have been described as warming the most rapidly^15^, where a decline in winter ice cover has been reported to result in an earlier onset of thermal stratification and, thus, an increase in the period over which the lake warms^20^. However, a direct cause and effect relationship of winter ice cover on summer LSWTs has yet to be reported, and its influence in driving excess summer LSWT warming is in question^21^. Moreover, other studies have demonstrated that ice cover is not a prerequisite for accelerated summer LSWT warming^12, 22^. Milder winter conditions, together with increased SAT and solar radiation in spring, resulting in earlier onset of stratification and thus increased heat absorption by surface waters^21, 23^, is now believed to be one of the main driving mechanism of rapid LSWT warming.

The relative timescales at which surface waters can react to equilibrating surface heat fluxes can influence a lake’s response to antecedent winter/spring conditions. Previous studies in the North American Great Lakes have demonstrated that the influence of thermal anomalies may only persist for a sufficient time to influence summer LSWT in very deep lakes, as these systems constantly adjust towards equilibrium with the atmosphere^24^. Others suggest that both latitude and depth could regulate the magnitude of accelerated warming^21, 25^ and recent studies illustrate that high latitude and high elevation lakes display substantial warming of their surface waters^6^.

In this contribution, we investigate the influence of early-warming season thermal anomalies on summer (July-September) LSWT for a global distribution of lakes (Table S1), characterised by varying mean depths and situated across climatic zones. Specifically, using satellite-derived LSWTs from 144 lakes (see Methods), we assess what are the relative contributions from changes in early-season conditions vs. changes in summer meteorological conditions to the amplification of summer LSWTs and how these factors co-vary along gradients of geographic location (latitude and elevation) and lake depth.

## Results and Discussion

**Figure 1:**
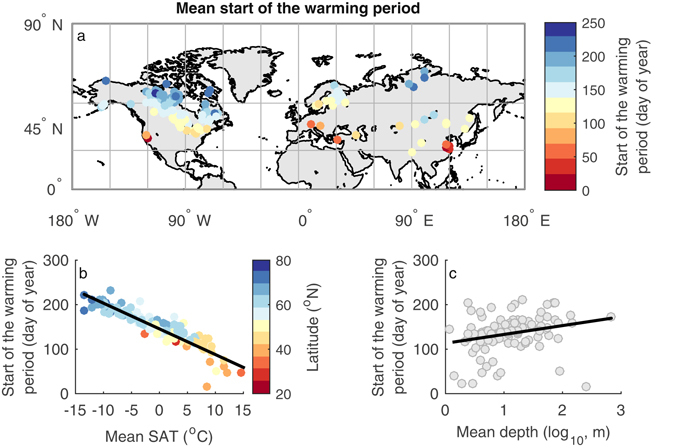
(**a**) Average first day of year in which lake surface water temperature (LSWT) persistently exceeds 4 °C (“start of the warming period”), and its relationship with (**b**) annual mean surface air temperature (SAT) and (**c**) lake mean depth. Colours in panel (**b**) signify latitude. Linear regressions of the statistically significant (p < 0.05) relationships are shown. The map was generated using the MATLAB mapping toolbox^43^ (URL-https://www.mathworks.com/products/mapping.html).

To investigate the influence of antecedent winter/spring conditions on summer (July–September) LSWT, we first calculate, for 144 global lakes, the day of year (DOY) in which LSWT first persistently exceeds 4 °C (close to the temperature of maximum density of freshwater), a marker used to capture the timing of the early phase of seasonal lake warming^26^. In this analysis, we refer to this DOY as the start of the warming period. A multiple linear regression model including latitude, elevation, and mean depth explains 69% of the variation in the start of the warming period across all lakes. Replacing latitude and elevation with annual mean SAT (to which latitude and elevation are related), results in a multiple linear regression that explains 84% of the variation in the start of the warming period (Table S2), with dependencies that are reasonably linear and statistically significant (Fig. 1). Throughout the analysis we use annual mean SAT to summarize the environmental conditions of the analysed lakes.

**Figure 2:**
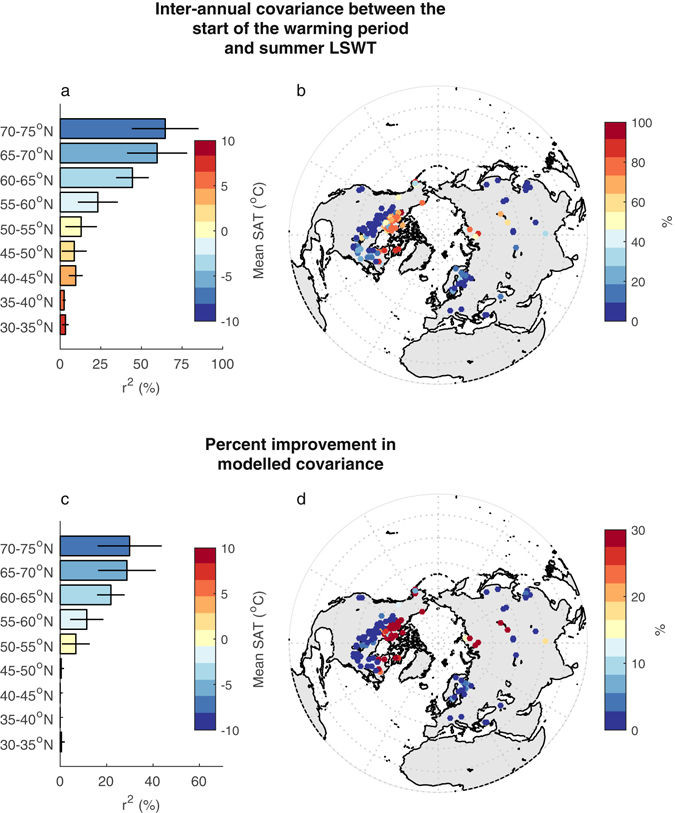
Relationship between (**a**,**b**) the proportion of variance (r^2^, %) shared between the inter-annual variability in summer lake surface water temperature (LSWT) and the inter-annual variability of the first day of year when LSWT persistently exceeds 4 °C. (**c**,**d**) The improvement (%) in the proportion of variance explained by introducing the date in which LSWT first persistently exceeds 4 °C to a multiple linear regression model of summer LSWT and summer surface air temperature (SAT) as predictors. Bar plots demonstrate averages for 5° latitudinal ranges, with the standard deviation indicated by the line. Colours in the bar plots signify the annual mean SAT for each latitudinal range. Maps were generated using the MATLAB mapping toolbox^43^ (URL-https://www.mathworks.com/products/mapping.html).

The start of the warming period can have a considerable influence on summer LSWTs as a result of the nonlinear response which occurs when LSWT crosses the 4 °C threshold, which for some lakes can be used to describe the timing of the onset of stratification^20, 27^. In stratifying lakes, the depth of the upper mixed layer changes from roughly the full lake depth and tends towards the shallower summer mixing depth after the 4 °C threshold is exceeded. LSWT warms more rapidly when the volume of water that directly participates in surface heat exchange is small. LSWTs can thus display an amplified response to SAT variability after LSWT crosses from <4 °C to >4 °C^21, 23^. To quantify the statistical influence of the start of the warming period on summer LSWT variability we calculate the proportion of variance (r^2^) shared between its inter-annual variability and that of summer LSWT for each lake (see Methods). By calculating the inter-annual variability in the start of the warming period, we determine if a lake has started to warm earlier or later compared to its climatological annual cycle. We find that the influence on summer LSWT of the anomaly in the start of the warming period varies systematically at a global scale (Fig. 2a,b), and is greater in colder regions (e.g. high latitudes).

To establish further the association of the start of the warming period and summer LSWT, we evaluate three linear regression models. These regressions consider the influence of two previously published predictors of summer LSWT, namely the summer-mean (July–September) and winter-mean (January–March) SAT^15^, in addition to considering the influence of the start of the warming period. The first regression model (model 1) relates the inter-annual summer LSWT to summer SAT only. Across all the lakes, the median of the explained inter-annual variability for model 1 is 69.6%, the maximum r^2^ for an individual lake being 89.8% and the minimum being 14.7%. The addition of winter SAT (model 2) has negligible influence on r^2^ (now expressed as an adjusted r^2^, ${{\rm{r}}}_{{\rm{adj}}}^{2}$, to account for the additional predictor) for 64% of lakes, and typically results in a change in the order of <10%. In Fig. 2c and d, we show the improvement in ${{\rm{r}}}_{{\rm{adj}}}^{2}$ over model 1 of adding the date in which the warming period starts (model 3) as an additional predictor (instead of the winter-mean SAT). We find that the explained variability of summer LSWT is more significantly increased when the start of warming period is included for lakes in cold regions (i.e. low mean SAT) and for deep lakes (Table S3).

**Figure 3:**
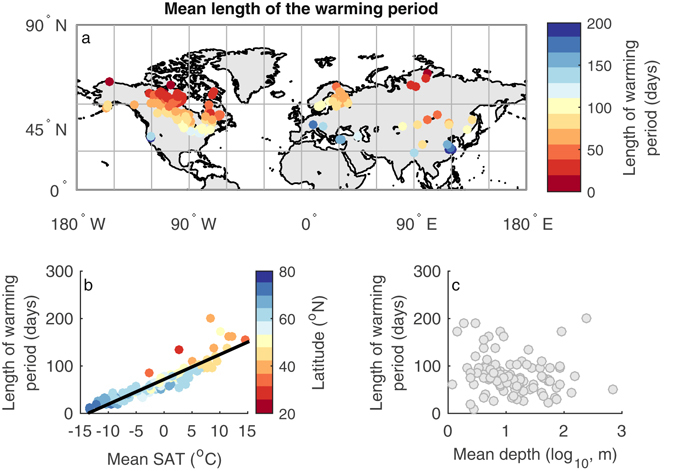
(**a**) Average length of the warming period, defined as the time difference (in days) between the average earliest day of year in which lake surface water temperature (LSWT) persistently exceeds 4 °C and the time of maximum temperature, and its relationship with (**b**) annual mean surface air temperature (SAT) and (**c**) mean depth. Colours in panel (**b**) signify latitude. Linear regressions of the statistically significant (p < 0.05) relationships are shown. The map was generated using the MATLAB mapping toolbox^43^ (URL-https://www.mathworks.com/products/mapping.html).

The mean SAT affects the degree of influence of the timing of the seasonal lake warming on LSWT because of the association (Fig. 3 and Table S4) between mean SAT and the length of the warming period (which we define as the number of days between the climatological start of the warming period and time of maximum LSWT). In colder regions, the duration of warming is shorter, making it more likely that anomalies in the timing of the start of warming can affect the maximum LSWT attained.

**Figure 4:**
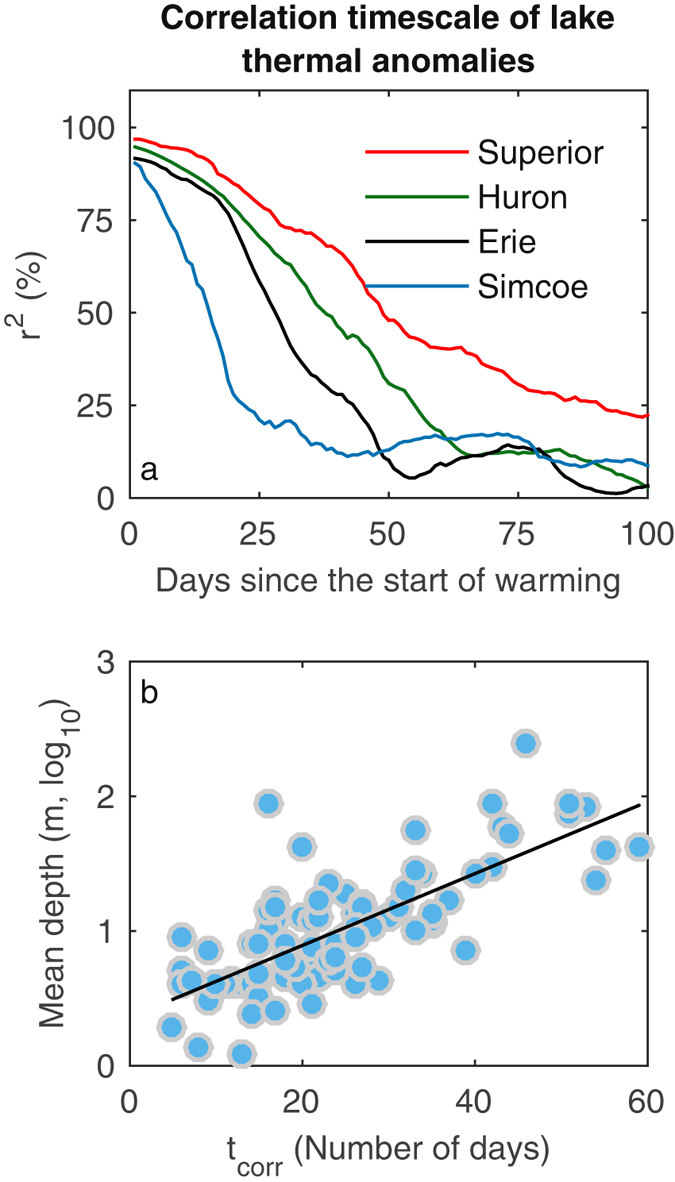
The covariance (r^2^) between the inter-annual variability in the start day of year (DOY) of the warming period and lake surface water temperature (LSWT) anomaly for 100 subsequent days for four North American Lakes (Superior [red, mean depth = 149 m]; Huron [green, mean depth = 59 m]; Erie [black, mean depth = 19 m]; and Simcoe [blue, mean depth = 15 m]). (**b**) Relationship between mean lake depth and the length of time over which anomalies in lake surface water temperature (LSWT) and the start of the warming period correlate significantly, t_corr_, for 144 lakes.

To demonstrate the significance of this association we calculate, for each lake, the correlation between the anomaly in the start of the warming period and later anomalies in LSWT (see Methods for details). In this way, we evaluate how LSWTs are influenced by the preceding early phase of lake warming and how this association varies with time. We find a sharp decrease in the correlation between these anomalies with time (e.g. Fig. 4a) – i.e. a decrease in the influence of the DOY of the start of warming on LSWT as time goes on. To quantify this, we calculate for each lake the duration over which these anomalies correlate significantly, t_corr_. This represents the period over which variations in the timing of the warming period can influence LSWT. Typically, this is during the first one or two months of a given lake’s climatological warming period, and thereafter, these anomalies are decorrelated by weather variability. This means that summer LSWTs can be influenced by the start of the warming period, and thus winter/spring conditions, only for lakes where the warming period is short relative to the persistence of early season thermal anomalies – i.e. where the length of the warming period is less than or comparable to t_corr_. Summer LSWTs are less influenced by the time at which the warming period starts if the length of warming period is much longer than t_corr_, as generally occurs equator-ward of 50 °N.

Mean depth influences the observed t_corr_ across the 144 lakes. In deeper lakes, the anomalies in the start of warming DOY and LSWT remain correlated for longer (Fig. 4a and b). This slower decrease in correlation over time results in a greater potential for variations in the start of the warming period to influence the subsequent summer LSWT. Therefore, winter/spring weather conditions and early-season thermal anomalies are more able to influence the summer LSWT of deep lakes; day-to-day changes in SAT erode established thermal anomalies more slowly in deep lakes^28^. In deep lakes that stratify, the transition in mixing depth (from maximum lake depth to the summer mixing depth) is a key factor in determining the effective heat capacity of the lake and the amplified response to SAT variability.

**Figure 5:**
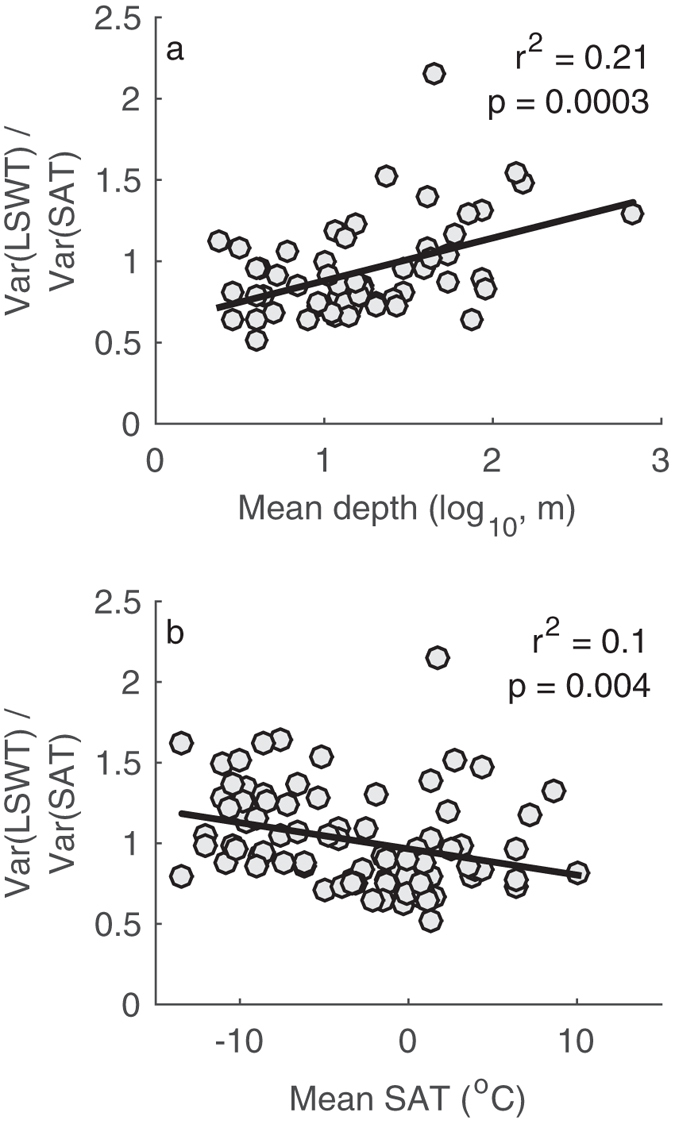
Comparison of the ratio of variances in the inter-annual variability of summer lake surface water temperature (LSWT) and surface air temperature (SAT) with (**a**) mean depth and (**b**) annual mean SAT. Linear regressions of the statistically significant (p < 0.05) relationships are shown.

To summarize the above analyses, we evaluate the influence of mean SAT and mean depth on summer LSWT variability relative to summer SAT variability. For each lake, we calculate the ratio of these inter-annual variances, Var(LSWT)/Var(SAT). Where this ratio exceeds 1, it is plausible to expect amplification of the summer LSWT response to summer SAT variability. The ratio of the variances is plotted against mean depth (Fig. 5a) and against annual mean SAT (Fig. 5b). The ratio of variances is significantly correlated with mean depth and is higher for deeper lakes (Fig. 5a). Our interpretation is that thermal anomalies are more likely to persist in deep lakes and enhance the summer LSWT variability. For this reason, in a scenario of year-round warming of SAT, deep lakes are more likely to show amplification of summer LSWT changes. The influence of annual mean SAT on amplification is also evident from looking at the ratio of variances against mean SAT (Fig. 5b). Mean annual SAT influences amplification in conjunction with mean depth via its association with the length of the warming period. Mean SAT is a critical factor for the length of the warming period relative to persistence time-scale for the lake, t_corr_. In terms of sensitivity to climatic variations, Fig. 5 suggests that where a lake is sufficiently deep (and tends to have a long t_corr_) and is situated in a cold climate (and tends to have a short warming period), amplification of summer LSWT to summer SAT variability is more likely to occur.

## Conclusions

We have analysed 20 years of satellite observations to study the influence of early-season thermal anomalies on summer LSWT in an analysis of 144 lakes across the globe. Our results indicate the relative importance of factors contributing to the amplified response of lakes to atmospheric temperature variability: where the length of the warming period is sufficiently short (for the depth of the lake in question), the direct effect of summer SAT forcing may be augmented by the persisting effect of an earlier start to the warming period. Timing of the warming period will have a greater influence on summer LSWT in lakes where the number of days between the start of warming and peak temperature is short and lake thermal inertia is large, which is associated with greater persistence of any temperature anomalies induced by an earlier start of the warming period. Thus, deep lakes situated in cold climates are most likely to display an amplified response to inter-annual SAT variability. Further analysis and longer data records are required to assess the degree to which similar mechanisms could drive an amplified response to SAT trends over the longer term associated with climate change. Amplified inter-annual summer LSWT variability can have a substantial influence on lake ecology. For example, warmer LSWTs can result in the modification of the biochemical compositions of some algal species^29^, result in advanced zooplankton phenology and reduced phytoplankton biomass^30^, promote the occurrence of toxic cyanobacterial blooms^31–33^, and threaten water quality^34^. Any climatic modification of SAT and LSWT variability has implications for local economies that depend on lakes for drinking water, agricultural irrigation, recreation, and tourism. Better understanding of the amplified response of LSWT to climatic variability, and the factors that control it, is therefore beneficial for climate-change impact and water management studies.

## Methods

### Lake surface temperature observations

In this study, we utilize LSWTs from the ATSR (Along Track Scanning Radiometer) Reprocessing for Climate: Lake Surface Water Temperature and Ice Cover (ARC-Lake) dataset^35^. LSWT observations are available for 246 globally distributed lakes, derived from the ATSR series, which consists of ATSR-1 (1991–1996), ATSR-2 (1995–2002), and AATSR (2002–2011), and retrieved at a spatial resolution of ~1 km at nadir and then averaged to 0.05° cells, where each 0.05° cell has an uncertainty in the order of 0.4 K (relative standard deviation).

In the ARC-Lake dataset, a target lake is identified on the basis of the geographical coordinates of a pixel in the ATSR imagery. A land/water mask reconciling the global lakes and wetlands database polygon area and the Naval Oceanographic Office data was developed specifically to define lake boundaries used in the ARC-Lake project^35^. Valid LSWTs are estimated only for pixels that are effectively free from cloud, where an algorithm based on Bayes’ theorem^36^ was used for assigning a clear-sky probability. The effectiveness of the lake product retrieval algorithms is assessed using two methods of data validation: analysis of the performance for case study images at full ATSR resolution and quantitative point comparisons with *in situ* observations. A match-up dataset from *in situ* temperature data consisting of 52 observation locations covering 18 lakes was constructed^35^. The mean differences ranged between the instruments from −0.34 to −0.09 °C (day) and −0.18 to +0.06 °C (night). Further details of the retrieval process and sensor specifications are provided in ref. 35.

In this study we use lake-mean surface water temperature time series, which are calculated for each lake by reconstruction (gap-filling), using dynamic empirical orthogonal functions^37^, of the whole-lake LSWT field from the intermittent and partial data coverage available from the satellite observations. We use lake mean surface water temperatures, which are calculated for each lake by averaging across the reconstructed area, providing a daily LSWT series. In this study we use night-time ARC-Lake LSWTs. As shown in the reference above, night-time LSWT retrievals are generally more accurate that those retrieved during the day, because near-surface variability from the skin effect is less^38^ and there is less near-surface stratification variability associated with variability in the solar radiation cycle^39^. In validation, the apparent daytime uncertainty (0.43 K) is greater than that at night (0.33 K) for a comparison to *in situ* data at particular locations, on average across the available validation locations and three satellite instruments^26^. The uncertainty in the night-time lake-mean values is difficult to quantify because the spatio-temporal correlations of error are not known, but the value of 0.33 K gives a plausible upper bound.

Of the 246 lakes in the ARC-Lake dataset, not all are suitable for inclusion in this study. We focus only on freshwater lakes, excluding lakes that are saline, which we define to be those with total dissolved solids content exceeding 3 g l^−1^. With this limit, we also only select lakes in which their surface temperatures cross from <4 °C to >4 °C during their climatological annual cycle. To calculate the climatological annual cycle, we average the LSWT for a given DOY across all complete years from the time-series. After these exclusions, 144 lakes remain. These lakes vary considerably in their geographic and morphological characteristics. They range in altitude between 0 m below sea level to 4446 m above sea level, in latitude between 28.97°N and 74.48°N, and in mean depth between 1.2 m and 680 m (Table S1).

### Lake depth information

Mean depth information for each lake included in this study was extracted from the Global Lake Database (GLDB, v2), for which data were either digitised from different topographic maps or extracted from ETOPO1, a 1 arc-minute relief model of Earth’s surfaces^40^.

### Air temperature observations

In this investigation we inform our LSWT analysis using SAT data gridded at 0.5° resolution from the Climatic Research Unit (CRU) time series version 3.23 (CRUTS v3.23)^41^. Data were selected for grid points situated closest to the lake centre and we selected periods that matched the available ARC-Lake data.

## Statistical Methods

### Regression models

A number of linear regression and multiple linear regression models were used in this study to evaluate the influence of geographic location and mean depth on summer LSWT variability. For all regressions where we compare the inter-annual variations in LSWT and SAT and/or the time in which LSWT first exceeded 4 °C, we first linearly detrended all time series to remove any biases which may emerge by comparing time series’ with similar trends. All statistical analyses in this study were performed in R^42^.

### Correlation of lake temperature anomalies

We calculate the timescale on which anomalies in the timing of the start of warming correlate significantly with LSWT anomalies, t_corr_, as follows. For a given lake, we find the latest start-of-warming DOY across all years in the time-series, and calculate anomalies in LSWT relative to that DOY until the time of LSWT maximum. We then calculate the covariance (r^2^) between the inter-annual variability in the start DOY of the warming period (i.e. the time in which LSWT first exceeded 4 °C) and the LSWT anomaly for each day after the latest start-of-warming DOY.

## Electronic supplementary material

Supplementary Information
