# Supplementary material for: Amplified surface temperature response of cold, deep lakes to inter-annual air temperature variability
Source: Sci Rep. 2017 Jun 23;7:4130. doi: 10.1038/s41598-017-04058-0 (PMC5482815; doi:10.1038/s41598-017-04058-0)
Supplement: Supplementary file 1 — Supplementary Information [file 41598_2017_4058_MOESM1_ESM.pdf]

Supporting information for

Amplified surface temperature response of cold, deep lakes to inter-annual air temperature variability

Author information

R. Iestyn Woolway[1*], Christopher J. Merchant[1]

Affiliation

1. Department of Meteorology, University of Reading, Reading, UK.

*Corresponding author: riwoolway@gmail.com

Contents of this file

Table S1 to S4

Table S1. General characteristics of the 144 lakes from which satellite-derived lake surface water temperatures were investigated in this study. Shown are the names of each lake, their latitude (φ), longitude (λ), altitude (h), and mean depth (z_{mean}).

Lake Name	φ (°N)	λ (°E)	h (m)	z_{mean} (m)
Superior	47.72	-88.23	184	149
Huron	44.78	-82.21	176	59
Michigan	43.86	-87.09	176	85
Baikal	53.63	108.14	450	680
Great Bear	65.91	-121.3	157	71.7
Great Slave	62.09	-114.37	158	41
Erie	42.25	-81.16	174	19
Winnipeg	52.12	-97.25	217	13
Ontario	43.85	-77.77	75	86
Ladoga	60.84	31.39	11	52
Onega	61.9	35.35	56	30
Athabasca	59.1	-109.96	212	26
Smallwood	54.19	-64.31	472	4.3
Reindeer	57.19	-102.27	337	17
Vanern	58.88	13.22	45	27
Winnipegosis	52.37	-100.05	252	4.24
Netilling	66.42	-70.28	30	20
Manitoba	50.99	-98.8	247	12
Nipigon	49.8	-88.55	283	54.9
Taymyr	74.48	100.76	24	2.8
Woods	49.38	-94.91	326	7.9
Khanka	44.94	132.42	64	4.5
Dubawnt	63.13	-101.44	235	
Peipus	58.41	27.59	29	7
Amadjuak	64.99	-71.13	112	
Cedar	53.33	-100.14	256	4.18
Hovsgol	51.02	100.48	1640	138
Iliamna	59.56	-154.9	35	44
Tai	31.21	120.24	2	1.9
Wollaston	58.3	-103.33	396	20.6
Hulun	48.97	117.38	539	5
Mistassini	50.82	-73.81	374	75
Nueltin	60.25	-99.4	277	
Vattern	58.33	14.57	91	39

Baker	64.13	-95.28	2	
Martre	63.33	-117.91	265	9
Hungtze	33.34	118.53	3	1.4
Astray	54.38	-66.32	557	
Kasba	60.34	-102.27	335	
Claire	58.59	-112.08	212	1.2
Yathkyed	62.69	-98.07	101	
Ronge	55.11	-104.83	371	12.7
Eau Claire	56.15	-74.4	251	
Rainy	48.61	-92.97	343	9.9
Sevan	40.39	45.29	1876	41
Vygozero	63.54	34.84	74	7.4
Cree	57.47	-106.64	484	14.9
Har Us	48.06	92.3	1191	4
Inari	69.04	27.83	126	14.4
Becharof	57.85	-156.4	22	56
Saint Clair	42.5	-82.73	174	3
Lesser Slave	55.43	-115.49	604	11.7
Red	48.04	-95.08	358	
Aberdeen	64.55	-98.59	76	
Bienville	55.05	-72.98	423	13
Paijanne	61.71	25.49	95	17
Saint Jean	48.66	-72.02	97	11.4
Beloye	60.18	37.64	122	4.15
Contwoyto	65.59	-110.66	444	
Malaren	59.44	16.19	18	11.9
Puruvesi	61.77	29.02	79	12
Champlain	44.45	-73.27	35	22.8
Wholdaia	60.69	-104.15	364	
Gods	54.62	-94.21	181	13.2
Island	53.85	-94.7	230	20.1
Takiyuak	66.28	-113.17	403	
Mackay	63.96	-111.3	426	
Topozero	65.62	32.09	112	16
Orivesi	62.35	29.59	79	17
Hottah	64.95	-118.44	221	
Pielinen	63.16	29.71	113	9.9
Nipissing	46.24	-79.92	212	4.5
Nonacho	61.82	-108.92	353	

Teshekpuk	70.59	-153.6	0	
Playgreen	54.07	-97.75	217	4
Haukivesi	62.1	28.52	80	17
Khantayskoe	68.36	91.18	54	73
Peter Pond	55.84	-108.55	423	13.7
South Moose	53.83	-100.04	256	4.1
Aylmer	64.15	-108.46	366	
Garry	65.95	-99.4	150	6.1
Segozero	63.32	33.76	129	29
Point	65.31	-113.84	464	
Chao	31.57	117.57	1	3
Simcoe	44.47	-79.42	233	15
Pyasino	69.77	87.78	17	4
Kaminak	62.2	-94.9	47	
Sasykkol	46.58	80.91	342	3.3
Cross	54.71	-97.58	203	8.2
Gras	64.54	-110.38	454	
Ennadai	60.96	-101.31	310	
Yamdrok	28.97	90.76	4446	23.6
Kamllukuak	62.28	-101.73	266	
Beysehir	37.78	31.52	1148	5
Tulemalu	62.99	-99.48	279	
Evans	50.97	-77.02	231	5
Pyaozero	66.07	30.98	118	15
Clinton Colden	63.94	-107.45	366	
Big Trout	53.77	-90.02	210	10.5
Dore	54.76	-107.28	459	10.9
Sakami	53.22	-76.75	174	8
Saint Joseph	51.04	-90.81	374	
Gaoyou	32.87	119.31	1	7.9
Buyr	47.81	117.69	597	8.1
Selwyn	60	-104.68	393	
Har	48.05	93.21	1160	4
Buffalo	60.22	-115.49	264	2.8
North Moose	54.05	-100.16	257	4.1
Balaton	46.88	17.83	126	4
Atlin	59.57	-133.75	704	86
Scott	60.02	-106.07	450	
Ferguson	69.41	-105.27	11	

Tathlina	60.54	-117.64	277	
South Henik	61.37	-97.29	184	
Kaminuriak	62.96	-95.79	149	
Churchill	55.96	-108.29	423	9
Angikuni	62.27	-100.04	256	
Deschambault	54.78	-103.45	329	6.2
Labaz	72.27	99.57	48	
Black	59.05	-105.73	277	
Artillery	63.17	-107.82	359	
Gyaring	34.92	97.27	4256	
Winnebago	44.02	-88.42	229	4.7
Ashuanipi	52.69	-66.14	596	
Keitele	62.89	25.99	107	7
Dauphin	51.27	-99.77	259	2.4
Constance	47.65	9.28	431	90
Payne	59.4	-73.82	138	
Sandy	53	-93.03	282	5.36
Granville	56.4	-100.21	259	
Snowbird	60.64	-102.94	358	
Mille Lacs	46.24	-93.65	381	6.4
Trout	60.58	-121.13	494	
Manouane	50.76	-70.99	494	
Tebesjuak	63.76	-98.98	144	
Naknek	58.64	-155.67	39	41
Tahoe	39.09	-120.04	1996	249
Egridir	38.07	30.85	946	9
Princess Mary	63.93	-97.66	115	
Weishan	34.61	117.24	7	4.2
Evoron	51.48	136.51	80	2.5
Pyhajarvi	61	22.28	52	5.5
Almanor	40.26	-121.19	1403	
Vesijarvi	61.09	25.39	96	6

Table S2. Summary output from a multiple linear regression model ($r^2 = 84\%$, $p < 0.01$) used to describe the global variations in the average time in which lake surface water temperature (LSWT) first persistently exceeds 4ºC.

Factor in model	Estimated coefficient	Standard error in coefficient	Significance
Constant	134.08	3.75	<0.001
Mean air temperature (°C)	-5.77	0.26	<0.001
Mean depth ($\log_{10}$, m)	10.90	3.07	<0.001

Table S3. Summary output from a multiple linear regression model used to describe the influence of mean depth and annual mean surface air temperature (SAT) on the improvement in the proportion of variance explained by introducing (a) winter (January to March) SAT or (b) the average time in which lake surface water temperature (LSWT) first persistently exceeds 4ºC, to a multiple linear regression model of summer (July to September) lake surface water temperature (LSWT) and summer SAT as a predictor.

		Estimated coefficient	Standard error in coefficient	Significance
**(a)**				
	Constant	-0.49	0.57	0.40
	Mean depth ($\log_{10}$, m)	0.52	0.46	0.26
	Mean air temperature (°C)	0.03	0.04	0.50
**(b)**				
	Constant	-2.22	2.70	0.41
	Mean depth ($\log_{10}$, m)	10.36	2.17	<0.001
	Mean air temperature (°C)	-1.11	0.19	<0.001

Table S4. Summary output from a multiple linear regression model used to describe the global variations in the length of the warming period, the time difference (in days) between the first time in which lake surface water temperature (LSWT) first persistently exceeds 4ºC and the time of maximum LSWT.

Factor in model	Estimated coefficient	Standard error in coefficient	Significance
Constant	72.21	3.84	<0.001
Mean air temperature (°C)	5.63	0.26	<0.001
Mean depth (log_{10}, m)	-2.27	3.14	0.47
